# Decoding the persistence of delayed hospital discharge: An in‐depth scoping review and insights from two decades

**DOI:** 10.1111/hex.14050

**Published:** 2024-04-17

**Authors:** Alyaa Abdelhalim, Manaf Zargoush, Norm Archer, Mehrdad Roham

**Affiliations:** ^1^ Information Systems, DeGroote School of Business McMaster University Hamilton Ontario Canada; ^2^ Health Policy & Management, DeGroote School of Business McMaster University Hamilton Ontario Canada

**Keywords:** alternate level of care, caregivers, continuous process improvement, delayed discharge, scoping review, transitional care

## Abstract

**Objective:**

This article addresses the persistent challenge of Delayed Hospital Discharge (DHD) and aims to provide a comprehensive overview, synthesis, and actionable, sustainable plan based on the synthesis of the systematic review articles spanning the past 24 years. Our research aims to comprehensively examine DHD, identifying its primary causes and emphasizing the significance of effective communication and management in healthcare settings.

**Methods:**

We conducted the Preferred Reporting Items for Systematic Reviews and Meta‐Analyses extension for Scoping Reviews (PRISMA‐ScR) method for synthesizing findings from 23 review papers published over the last two decades, encompassing over 700 studies. In addition, we employed a practical and comprehensive framework to tackle DHD. Rooted in Linderman's model, our approach focused on continuous process improvement (CPI), which highlights senior management commitment, technical/administrative support, and social/transitional care. Our proposed CPI method comprised several stages: planning, implementation, data analysis, and adaptation, all contributing to continuous improvement in healthcare delivery. This method provided valuable insights and recommendations for addressing DHD challenges.

**Findings:**

Our DHD analysis revealed crucial insights across multiple dimensions. Firstly, examining causes and interventions uncovered issues such as limited discharge destinations, signaling unsustainable solutions, and inefficient care coordination. The second aspect explored the patient and caregiver experience, emphasizing challenges linked to staff uncertainty and negative physical environments, with notable attention to the underexplored area of caregiver experience. The third theme explored organizational and individual factors, including cognitive impairment and socioeconomic influences. The findings emphasized the importance of incorporating patients' data, recognizing its complexity and current avoidance. Finally, the role of transitional and social care and financial strategies was scrutinized, emphasizing the need for multicomponent, context‐specific interventions to address DHD effectively.

**Conclusion:**

This study addresses gaps in the literature, challenges prevailing solutions, and offers practical pathways for reducing DHD, contributing significantly to healthcare quality and patient outcomes. The synthesis introduces the vital CPI stage, enhancing Linderman's work and providing a pragmatic framework to eradicate delayed discharge. Future efforts will address practitioner consultations to enhance perspectives and further enrich the study.

**Patient or Public Contribution:**

Our scoping review synthesizes and analyzes existing systematic review articles and emphasizes offering practical, actionable solutions. While our approach does not directly engage patients, it strategically focuses on extracting insights from the literature to create a CPI framework. This unique aspect is intentionally designed to yield tangible benefits for patients, service users, caregivers, and the public. Our actionable recommendations aim to improve hospital discharge processes for better healthcare outcomes and experiences. This detailed analysis goes beyond theoretical considerations and provides a practical guide to improve healthcare practices and policies.

## 1 INTRODUCTION

As the global population ages, healthcare systems in developed countries face increasing pressures to meet demand and manage costs. The United States has witnessed a surge in healthcare demand due to aging and chronic diseases, leading to growing costs.^1^ The rise in chronic diseases, at 4.2% annually, contributes significantly to increased demand.^2^ As the aging population grows, healthcare costs rise, making cost management crucial for quality patient care.^3^, ^4^ Delayed Hospital Discharge (DHD) is a critical issue that occurs when patients ready for discharge remain in hospitals due to a lack of external care facilities.^5^, ^6^ This issue, known by various terms globally, prolongs hospital stays, increases costs, reduces capacity, and leads to poorer patient outcomes. Therefore, addressing DHD's root causes and effects is essential.^5^, ^7^, ^8^, ^9^, ^10^, ^11^

The literature on healthcare management and patient outcomes offers insights into healthcare system challenges. Åhlin et al. identified barriers to hospital patient flow, suggesting a need for further research.^12^ Glasby et al. and Bhatia et al. highlighted DHD issues in the United States and Canada, respectively,^13^, ^14^ calling for systemic solutions and policy changes. Micallef et al. explored DHD in acute settings, emphasizing the need for practical policy implications.^5^ Philp et al. reported on interventions to reduce hospital bed use among frail older people.^15^ Meanwhile, van Sluisveld et al. evaluated clinical handover quality at patient discharge, noting the need for better research methods.^16^ Modas et al. assessed tools for predicting prolonged hospital stays, with a gap noted in practical healthcare policy implications.^10^ Plante et al. linked cognitive impairment to longer hospital stays, suggesting the need for strategies to address the issue.^8^ Lin et al. focused on contributing factors to the ICU discharge,^9^ and Rameli and Rajendran reviewed the effectiveness of multidisciplinary discharge planning teams.^17^ Peltonen et al. looked at organizational factors in critical care settings, identifying a need for more generalizable evidence.^18^ Bradley et al. and Fox et al. examined discharge planning's effectiveness, with a call for understanding study limitations and providing clearer policy implications.^19^, ^20^ McGilton et al. provided insights into transitional care programs (TCPs) for older adults with DHD, emphasizing the need for more research.^21^ Spiers et al. examined social care's impact on healthcare utilization, identifying a need for comprehensive evidence.^22^ Everall et al. and Rojas‐García et al. analysed DHD experiences from patient and caregiver perspectives, highlighting the need for more holistic approaches and practical recommendations.^23^, ^24^ Cadel et al. reviewed DHD initiatives, calling for more thorough patient experience reporting.^6^ Landeiro investigated DHD costs, advocating for standardized methods and patient‐centred approaches.^3^ Mason et al. considered integrating health and social care funds, emphasizing the need for careful planning.^25^

It is evident that the DHD literature lacks consensus on its causes and outcomes. This study systematically reviews review articles from the past decade to understand perspectives and identify primary reasons and contributing factors. Scoping reviews focusing on published systematic reviews offer benefits, helping policymaking by providing an overview of existing research and identifying knowledge gaps. Published systematic reviews offer insights from multiple studies, providing a broader perspective on DHD factors. Their methodological rigour ensures reliable evidence for policy recommendations, facilitating evidence‐based decision‐making and providing efficient guidance for healthcare systems to address DHD.

## 2 MATERIALS AND METHODS

This study utilized the PRISMA‐ScR method, a structured approach for conducting scoping reviews, ensuring transparency and rigour in methodology,^21^, ^26^ and enhancing the reliability and credibility of our study outcomes. Following the five stages outlined by Levac et al.,^27^ we identified the research question, relevant literature, selected studies for inclusion, organized data, and summarized results. Leveraging the Cochrane Review of Systematic Reviews, our scoping review provides evidence‐based insights for healthcare decision‐making, integrating findings from multiple studies.^28^ By presenting a comprehensive summary, we aim to bridge the research‐to‐practice gap and potentially enhance patient outcomes. Our scoping review aims to clarify the current state and identify ways to improve DHD solutions.

### 2.1 Identifying research questions

Despite extensive research, DHD remains problematic, with worsening consequences for patients and healthcare systems. The scoping review was guided by two specific research questions: (1) What are the primary causes and contributing factors that aggravate the DHD problem? and (2) Which interventions and funding programs have alleviated the DHD issue?

### 2.2 Identifying relevant studies

To investigate the impacts of DHD, we searched electronic databases for systematic (or scoping) reviews published in English from 2000 until 31 January 2024. This timeframe ensures relevance to current healthcare systems by focusing on literature post‐2000, reflecting significant evolutions in healthcare management. Databases used for this search included Scopus, Web of Science, EMBASE (Ovid interface), CINAHL, and PubMed. The search strategy involved keywords related to hospital discharge delays, as follows: (‘delayed discharge’), (‘alternate level of care’), (‘inappropriate use of bed’), (‘inappropriate hospital use’), (‘inappropriate acute bed use’), (‘inappropriate bed use’), (‘discharge delay’), (‘bed block*’), (‘social admission’), and (‘delay* transfer’). English‐language and review articles were included after applying filters to streamline search results. Reference lists from identified articles were examined for further relevant studies. Google Scholar was used to capture grey literature, including resources like technical reports. Covidence® is a web‐based tool for systematic reviews that helped us to minimize bias by organizing search results, facilitating full‐text screening, and data extraction. Articles that passed the initial screening were further evaluated for their relevance and quality based on the set inclusion criteria. The final selection comprised 23 articles, from which one author systematically extracted and analysed data to address the research question.

### 2.3 Study selection

Our inclusion/criteria encompassed systematic review articles on DHD from the past two decades. Exclusion criteria were nonsystematic reviews, non‐English articles, and studies not centrally focused on DHD (Table 1). We did not restrict our search by patients' discharge destinations, as this would narrow the scope of factors explored for DHD. This broad criterion ensured an inclusive analysis of diverse factors impacting DHD. Detailed inclusion/exclusion criteria for the 23 selected articles are outlined in Table C2.

**Table 1:** The inclusion and exclusion criteria for this study.

	Inclusion	Exclusion
Language	English	Other languages
Year	2000–31 January 2024.	Before 2000
Article Type	Review (systematic, or scoping)	Narratives, essays, not a review
Topic	Delayed hospital discharge, alternate level of care patients and their synonyms^a^	Topics that do not study delayed hospital discharge or alternate level of care patients
Others	ALC patients from acute care	Emergency Department, nonacute patients

a As mentioned in the keywords used for the search engines.

### 2.4 Charting the data

Data extraction from Covidence® to a Microsoft Excel Workbook was employed for charting purposes. The charted data encompassed details such as the primary author, publication year, article title, study objectives, and inclusion/exclusion criteria. These data are presented in Tables 2, 3, 4, 5, and the comprehensive details can be found in Tables C1 and C2.

**Table 2:** Characteristics of the Articles of Topic 1.

References	Articles reviewed	Reasons for DHD	Key findings
Luis et al.^29^	13 2001–2022	1. Multifactorial Issues influenced by factors internal or external to hospitals. 2. The medical characteristics of the patients. 3. Family refusal of home‐based care and lack of alternative care centres. 4. The complexity of patients’ processes was linked to delayed discharges. 5. Certain medical conditions, such as acute cerebrovascular disease. 6. 6‐ Inadequate social care and the inability to balance the care the patient needs.	1. The review highlighted the prevalence and average duration of delayed discharge and the potential negative repercussions on patients’ health and well‐being. 2. The study identified the limited literature available on delayed discharge in Spain.
Åhlin et al.^12^	92 2010–2020	1. Long lead times. 2. Inefficient capacity coordination 3. Inefficient capacity 4. Large capacity utilization variation 5. Inefficient capacity utilization 6. High work in the process 7. Inefficient patient process transfer 8. Inefficient support process transfer 9. Unpredictable inflow variation 10. Changing demand 11. Inefficient outflow process 12. Low interorganizational coordination	1. Several root causes are more easily addressed and can lead to capacity improvements without increasing expenditures. 2. Recommended to use of improvement agents and healthcare managers at hospitals
Goncalves‐Bradley et al.^19^	33 2015–2022	1. Incomplete assessment. 2. Disruption of care arrangements. 3. Difficulty accessing follow‐up care. 4. Poor communication.	Personalized discharge planning interventions may significantly impact hospital LOS and readmission rates for patients, particularly elderly with medical conditions. However, the evidence on health status and costs remains limited.
Rameli et al.^17^	27 2001–2021	1. frailty 2. multimorbidity 3. Lack of information sharing with patients and caregivers 4. Medication errors 5. Lack of community coordination with general practitioners 6. Lack of follow‐up	1. There were no specific follow‐up studies on outcomes after the intervention. 2. Effective communication between hospital teams and primary care teams enables continuity of care post‐discharge. 3. Greater emphasis must be placed on frailty scoring to guide the discharge destination.
Micallef et al.^5^	64 1990–2019	1. Conflicts of interest between health professionals. 2. procedural delays. 3. Confusion/redundancy in the care plan. 4. Lack of discharge planning and poor communication 5. Insufficient statutory services.	DHD cause: 1. Severe accident and emergency. 2. Overcrowding and bed‐blocking. 3. inevitable negative financial implications.
Tipton et al.^30^	19 2010–2017	1. Healthcare disparities. 2. Medically complex needs. 3. Environmental Factors: including unique resources, personnel, and leadership. 4. Admission Process and Discharge Disposition. 5. Structural Interventions. 6. Social and Economic Barriers.	1. The report highlights that rigorous systematic reviews have not consistently demonstrated the effectiveness of strategies to reduce length of stay. 2. The report identifies research gaps, including the lack of hospital detail. 3. Health system leaders, researchers, and policymakers are urged to collaborate to develop informed strategies for LOS reduction.
Cadel et al.^6^	66 2004–2019	Not mentioned	1. Adding more intermediate care beds alleviates pressures in acute care in the short term. 2. Focus on optimizing patient and caregiver experiences and outcomes. 3. lack of information on implementation strategy, setting, and population characteristics. 4. inconsistency in how DHD was defined.
Bhatia et al.^14^	6 2016–2019	Not mentioned	1. A single approach may be insufficient to tackle the DHD/ALC challenge. 2. Attempts to reduce DHD/ALC rates should be multicomponent tailored to the local context. 3. Financial incentives alone are insufficient to reduce DHD/ALC rates.
Landeiro et al.^3^	64 1990–2015	1. At an individual level, the specific needs of certain patient groups remain unaddressed. 2. At an organizational level, delays in providing acute hospital services impact DHD. 3. At a structural level, there is poor communication between health care and social care.	1. Local factors play an important role in reducing DHD. 2. There is a need for internationally recognized parameters to determine when a patient is medically ready for discharge. 3. Other factors may be associated with the proportion of DHD, such as the level of social support of the patients, level of income, level of dependency on daily living activities, and comorbidities.
Goncalves‐Bradley et al.^31^	19 1946–2015	Not mentioned	Educating patients in the treatment group about medication and side effects might have made them more likely to visit the emergency department.
van Sluisveld et al.^16^	11 Up to 2013	1. Poor information transfer is a common patient safety issue in all types of handover settings 2. The use of an inappropriate intervention about the underlying healthcare problem 3. Measurement of inappropriate outcomes 4. Suboptimal research population, such as a low mortality rate at the baseline	1. Using an ICU discharge form is an effective intervention. 2. Effective handover is a timely transfer with complete information on the transferred patient.
Philp et al.^15^	48 2007–2013	Not mentioned	No evidence of impact on hospital bed use from interventions such as multifactorial fall prevention services, day hospital services, medication reviews, exercise programs in the community, nutritional enhancement in hospitals, and nurse‐led transitional care units.
Glasby et al.^13^	21 NA	1. Lack of rehabilitation. 2. Hospital‐based delays. 3. Awaiting transfer to another hospital/wider NHS services. 4. Assessment delays/uncertainty over aftercare. 5. Funding difficulties/delays. 6. Awaiting care home placement/availability. 7. Awaiting domiciliary/community package/lack of community services. 8. Staff shortages (various professions/agencies). 9. Housing/aids and adaptations/social. 10. Circumstances user/caregiver‐related factors	1. Different definitions of DHD raise concerns about the accuracy of official statistics. 2. The literature lacks a patient and caregiver perspective.

Abbreviation: DHD, Delayed Hospital Discharge.

**Table 3:** Characteristics of the articles of Topic 2.

References	Articles reviewed	Reasons for DHD	Key findings
Everall et al.^23^	7 1998–2018	1. Overall uncertainty. 2. The impact of hospital staff and physical environment. 3. Mental and physical deterioration. 4. Lack of engagement in decision‐making. 5. Initial disbelief followed by reluctant acceptance of the situation.	1. On an Interpersonal level, facilitating accurate and timely information sharing. 2. On a facility level, developing guidelines and training staff. 3. On the system level, creating policies and processes. 4. There was a lack of physical and mental health support during the delayed transition.
Rojas‐Garcia et al.^24^	37 2000–2016	1. Lack of personal privacy, tedium or boredom, depression, and loss of independence. 2. Addressing DHD was perceived by staff to dehumanize patients. 3. Some health professionals reported negative reactions towards patients. 4. Delays also undermined information sharing between health and social care.	1. Introduced a unique definition of DHD. 2. Findings provide renewed emphasis on the need to standardize measuring delays.

Abbreviation: DHD, Delayed Hospital Discharge.

**Table 4:** Characteristics of the articles of Topic 3.

References	Articles reviewed	Reasons for DHD	Key findings
Plante et al.^8^	58 1997–2019	1. A positive association between CI and LOS or DHD was reported. 2. The causes of hospitalization and co‐morbidities influence LOS or DD. 3. LOS or DHD is influenced by the initial living arrangement of a patient and the discharge destination.	1. Hospitals contribute to the functional decline experienced by patients, leading to an increase in the level of care needed when they return to the community. 2. Changes in bed supply may have mixed effects on LOS or DHD. 3. Patients who decline before being admitted to the hospital have a better recovery than those who deteriorated in the hospital.
Modas et al.^10^	7 2001–2017	Age and patient characteristics can predict the patients’ at‐risk for prolonged hospitalization.	Suggested further investigation, with new research protocols for possibly including new studies and identifying other instruments.
Peltonen et al.^18^	6	1. Poor Information management and teamwork. 2. Lack of shared situational awareness, such as Lack of resources, Busy workload (unit/hospital), and lack of an available bed. 3. Lack of adequate staff. 4. The receiving unit is not ready for transfer. 5‐ Time of discharge.	1. Explanatory research is scarce. 2. Patient admission and discharge delays are common problems. 3. Reducing ICU admission and discharge delays requires an interprofessional and multifactorial evaluation approach to the whole critical care process in the hospital. 4. Delayed discharge patients had a higher hospital mortality rate.
Fox et al.^20^	9 NA	Failure to plan discharge planning upon patient admission to the hospital.	1. Patients who did not receive early discharge planning experienced higher readmission rates and longer lengths of readmission hospital stay. 2. Compared to usual care, those who received early discharge planning had higher overall quality of life scores. 3. Implementing early discharge planning focused on functional needs assessment anticipates reductions in older adults’ hospital readmissions by 22%. 4. The goal of early discharge planning is to facilitate the transition of care back to the community.
Lin et al.^9^	21 Up to 2009	1. Lack of agreement in clinical decision‐making. 2. The ward staff's lack of knowledge and skills to look after the higher acuity patient. 3. Teams make fewer mistakes when each member understands their roles and responsibilities. 4. A multidisciplinary team involved in daily goal setting reduced adverse events. 5. Improved communication and collaboration among ICU doctors and nurses through team training decreased patient ICU mortality	1. The triage discharge model must be cautiously used to avoid premature discharge. 2. The lack of agreement in discharge decision‐making indicates a problem in staff training.

Abbreviation: DHD, Delayed Hospital Discharge.

**Table 5:** Characteristics of the Articles of Topic 4.

References	Articles reviewed	Interventions (if included)	Other improvement factors	Key findings
Mason et al.^25^	122 1999–2015	Integrated care with integrated financing	1. Improve access to care 2. Reduce unplanned admissions and readmissions 3. Increase community care. 4. Reduce total costs. 5. Improve outcomes. 6. Improve the care quality. 7. Reduce the length of stay. 8. Reduce residential care. 9. Improve patient and user experience of care.	1. Failed to integrate acute and long‐term care. 2. The difficulty of engaging with those eligible for care. 3. Challenges of implementing a fully operational information management and technology system. 4. No scheme demonstrated a sustained reduction in hospital use.
Spiers et al.^22^	12 1946–2019	Availability of social care	Not mentioned	1. Higher expenditures on social care are associated with fewer readmissions and DHD. 2. Social care reduces the demand for secondary health services.
McGilton et al.^21^	37 Up to 2019	Transitional care programs	NA	1. Unclear on how family members are involved in discharge planning. 2. The literature lacks the benefits of integrating comprehensive geriatric assessments. 3. Account for social vulnerabilities, including discharge planning. 4. Better acute care practices can occur upstream instead of focusing on repairing losses that occurred in hospitals.

### 2.5 Quality assessment and risk of bias

Quality domains like Selection, Information, and Analysis biases are crucial in intervention studies. Our review, focusing solely on published systematic reviews, inherently excludes unpublished studies, reducing publication bias. Selective outcome reporting and ascertainment biases are not applicable due to our use of peer‐reviewed journals. We made sure to clearly list our inclusion and exclusion criteria in a transparent manner to minimize the risk of bias. Information bias was minimized by disregarding authors' names, journal ranks, and study locations during screening. English language restriction is unlikely to introduce systematic bias. Analysis bias is addressed through graphs and charts detailing research evolution without biasing results, as shown in Section 4.

## 3 BIBLIOMETRIC ANALYSIS

Our scoping review utilized bibliometric analysis with Biblioshiny® to track DHD's thematic trends in healthcare research. Bibliometric analysis is critical for identifying research topics, authors, and field knowledge. It complements systematic reviews by mapping the scientific landscape and revealing trends. Thematic mapping adds structure by illustrating keyword connections, helping to spot research patterns and gaps. Biblioshiny®, an R Studio®‐powered tool, processes bibliographic data from databases like PubMed and Scopus, enabling interactive exploration and visualization. We analysed metadata, including titles and authors, to identify trends, prolific contributors, and topic networks over time.

## 4 COLLATING, SUMMARIZING, AND REPORTING THE RESULTS (PRISMA)

**Figure 1:**
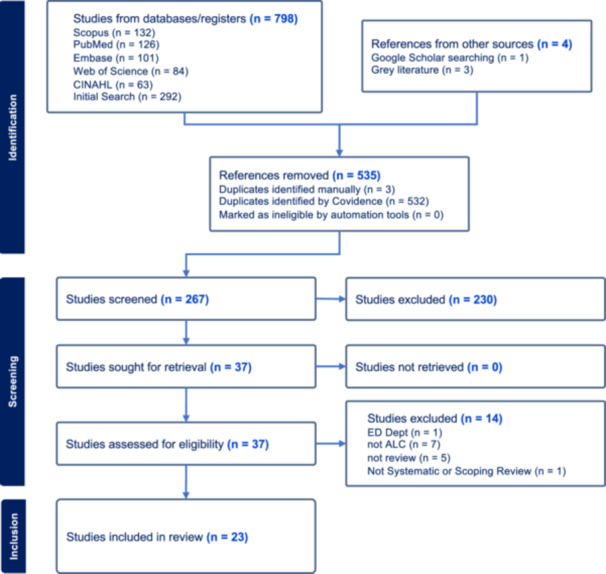
PRISMA flowchart of the selection process for literature review.

The initial search retrieved a total of 806 articles from databases, grey literature, and Google Scholar (Figure 1). After screening titles and abstracts, 535 duplicates were removed. Following inclusion and exclusion criteria, 230 articles were excluded, with an additional 14 articles after a full‐text review, resulting in 23 unique articles for synthesis. Inductive reasoning and thematic analysis categorized systematic reviews into topics, yielding four topics.

**Figure 2:**
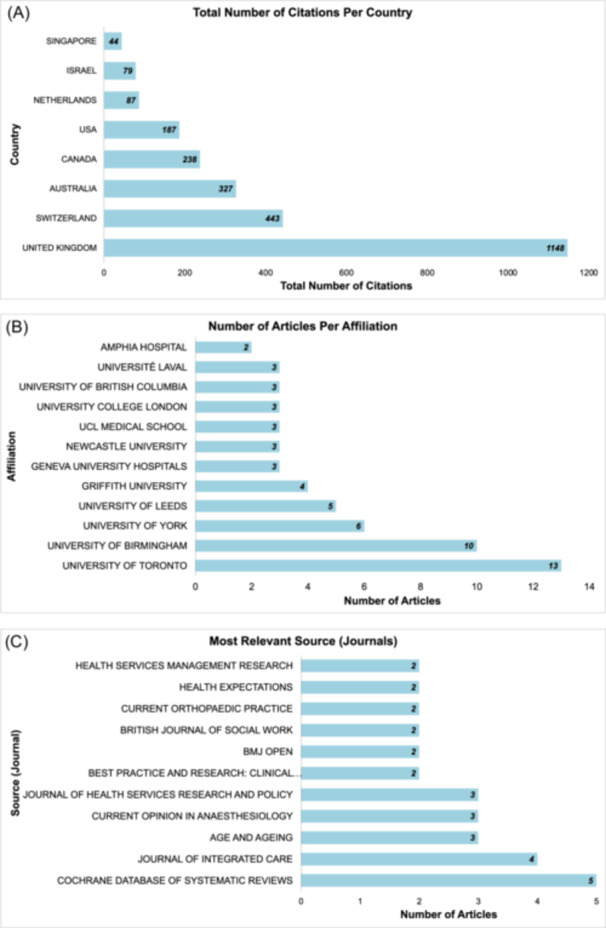
(A) Most cited countries, (B) most frequent (relevant) affiliations, and (C) most relevant publications source.

As the results in Figure 2 indicate, the United Kingdom, Switzerland, and Australia were the most cited countries for DHD. The University of Toronto was the leading affiliation, and the Cochrane Database of Systematic Reviews was the leading source for DHD research.

**Figure 3:**
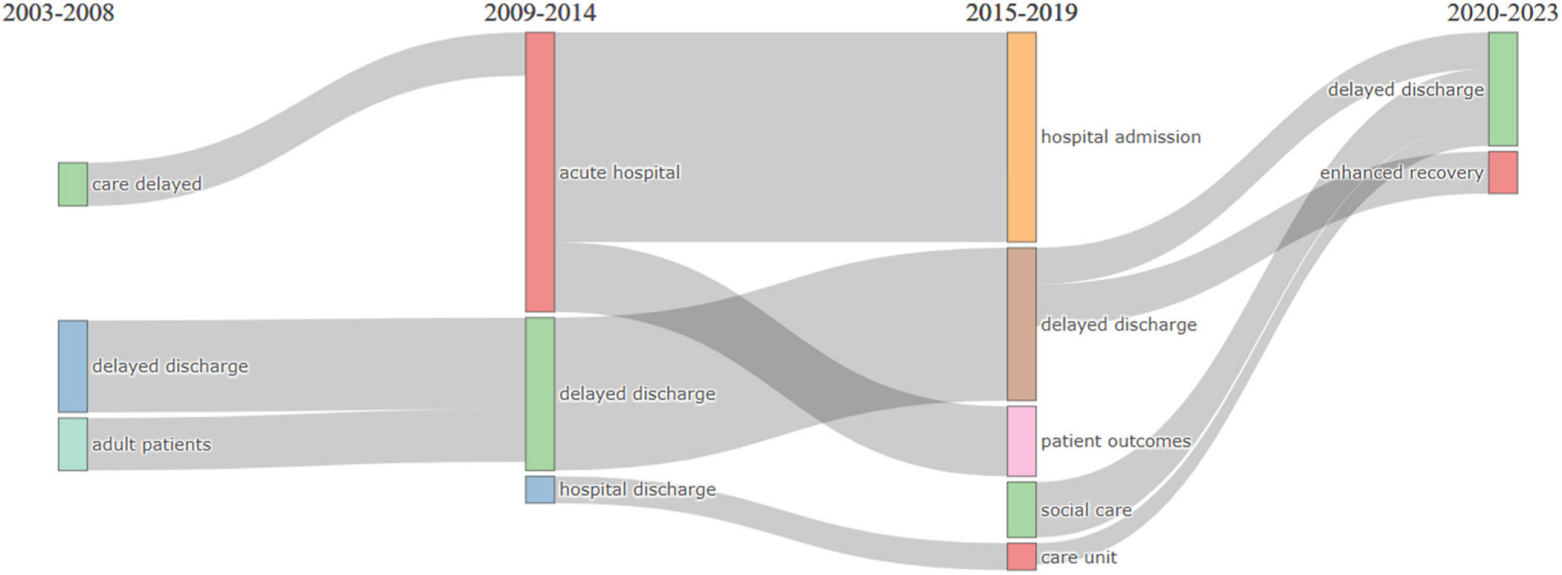
Evolution of the themes over time.

**Figure 4:**
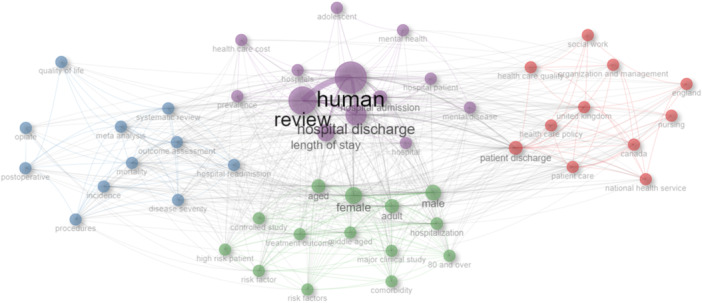
Words co‐occurrence in literature abstracts.

Figure 3 illustrates the thematic evolution from 132 Scopus articles on DHD, showing how frequent words in abstracts have shifted over time, reflecting evolving research contexts. Connections between themes demonstrate keyword development, convergence, or divergence, with recent trends highlighting social care, recovery, and patient outcomes. The persistent presence of ‘delayed discharge’ underscores ongoing challenges. Figure 4 visualizes word co‐occurrences, revealing DHD's impacts on the female population over two decades.

## 5 SYNTHESIS OF THE RESULTS

This section presents an analysis of 23 systematic reviews that identify four main topics that have dominated these articles over the past two decades. These topics are as follows (a complete summary is available in Table C1):

### 5.1 Topic 1: Causes (barriers) and interventions

An in‐depth scrutiny of Topic 1, encompassing 13 articles, is outlined in Table 2. Glasby et al.^13^ conducted a comprehensive review, identifying 21 documents that shed light on multifaceted causes contributing to DHD, ranging from systemic deficiencies like lack of rehabilitation services and funding hurdles to logistical challenges such as hospital‐based delays and assessment bottlenecks. Their discourse examined promising interventions like nurse‐led discharge planning and innovative strategies such as introducing discharge lounges and enhancements in local rehabilitation services, underscoring the need for collaborative efforts across health and social care sectors. Moreover, they astutely highlighted research gaps, particularly the oversight of addressing the unique needs of vulnerable demographics like older adults with mental health issues. Similarly, Luis et al.'s work in Spain to quantify DHD cases provided valuable insights, emphasizing the profound impact of clinical characteristics, especially among elderly, frail, and dependent individuals.^29^ Their review not only underscored the pressing need for interventions targeting inappropriate admissions and extended stays but also identified multiple‐component strategies tightly linked to discharge planning as effective avenues for DHD mitigation.

Tipton et al.'s report echoed concerns regarding medically complex patients facing heightened risks of discharge delays, elucidating the plethora of interventions designed to curtail hospital length of stay, albeit with inconsistent evidence, necessitating further scrutiny.^30^ Åhlin et al.'s identification of barriers to patient throughput within hospital settings elucidated pervasive challenges like long lead times and inefficient capacity coordination, alongside root causes such as staffing inadequacies and operational deficiencies, underpinning the imperative for systematic reforms.^12^ Similarly, Cadel et al.'s review elucidated an array of interventions encompassing practice changes, information‐sharing initiatives, and infrastructure enhancements, all geared towards expediting discharge processes and ameliorating delay‐related burdens.^6^ However, they expressed dissatisfaction with the lack of detailed insights into patient, caregiver, and provider experiences, urging more comprehensive investigations. Micallef et al.'s finding of organizational impediments underscored the pivotal role of strategic interventions such as developing discharge facilitation tools and ‘discharge before noon’ policies in streamlining discharge workflows.^5^ Meanwhile, Sluisveld et al.'s scrutiny of patient handover quality highlighted deficiencies in clinical practices and communication protocols, advocating for adopting liaison nurse roles and standardized handover protocols to enhance continuity of care and mitigate discharge‐related adversities.^16^

Philp et al.'s comprehensive analysis investigated the escalating demand for acute hospital beds, accentuating the necessity of interventions like care coordination and community‐based rehabilitation services to alleviate strain on hospital resources and ensure seamless transitions post‐discharge for frail older populations.^15^ Landeiro et al.'s examination of costs associated with DHD provided valuable economic perspectives, emphasizing the imperative for methodological standardization to facilitate comparative analyses and informed resource allocations.^3^ Rameli et al.'s emphasis on individualized care pathways underscored the significance of holistic approaches in optimizing discharge outcomes for older adults, stressing the pivotal role of multidisciplinary discharge planning teams.^17^ Similarly, Gonçalves‐Bradley et al.'s emphasis on personalized discharge plans highlighted the potential for tailored interventions in reducing hospital length of stay, albeit with acknowledgement of the need for robust evidence to drive effective implementation strategies.^31^ Gonçalves‐Bradley et al.'s updated review reiterated persistent causes of DHD, advocating for structured discharge planning and streamlined post‐discharge services as pivotal interventions to mitigate delay‐related adversities.^19^ Lastly, Bhatia et al.'s critique of existing approaches illuminated systemic challenges in tackling DHD, warranting more holistic, jurisdiction‐wide strategies to effectively address the DHD problem's multifaceted nature effectively.^14^

### 5.2 Topic 2: Patient and caregiver experience of DHD

Two systematic reviews shed light on the experiences of patients and caregivers affected by DHD, as detailed in Table 3. Everall et al.^23^ identified strategies to alleviate DHD, including providing timely information to patients and families, enhancing patient mobility, addressing long‐term care wait lists, and supporting couples. They recommended staff training on transitional and patient‐centred care, patient feedback avenues, and handover support. Confirming DHD as a system‐level issue, Rojas‐García et al.^24^ emphasized DHD's adverse impacts on patient well‐being and hospital experiences, citing anxiety, boredom, and loss of independence. Caregivers, comprising hospital staff, faced stress and pressure to expedite discharge. Transfer delays between healthcare providers fueled mistrust. Notably, caregiver definitions varied between studies: Everall et al.^23^ defined them as unpaid family or friends aiding patients, while Rojas‐García et al.^24^ referred to healthcare and social care practitioners. Standardizing caregiver definitions is crucial for consistent research. Existing literature lacks comprehensive investigation into caregiver experiences, necessitating a unified approach for accuracy.

### 5.3 Topic 3: Organizational and individual factors that impact DHD

The review articles on DHD in this topic studied its impact considering both organizational and individual factors (Table 4). Individual factors encompass professional and medical staff roles and, to some extent, patient characteristics. Lin et al.^9^ delineated patient factors within their framework but could not investigate them due to their complexity. Organizational factors include resource availability, discharge policies, and interventions like ICU liaison nurses, which enhance ICU performance, reduce hospital stays, and lower patient mortality. Individual factors, like clinical decision‐making discrepancies and nurses' proactive bed management, were emphasized.

Fox et al.^20^ linked discharge planning in an early admission stage with fewer hospital readmissions and shorter readmission lengths post‐index discharge. However, they found no significant differences in initial hospital stays, mortality rates, or discharge planning satisfaction. Peltonen et al.^18^ identified delays in 38% of admissions and 22–67% of discharges, mainly due to organizational issues like information management and resource scarcity. Strategies like direct admissions to coronary care units and ICU liaison services alleviated ICU discharge delays. Barriers included the limited number of reviewed articles, insufficient research on admission delays, and inconsistent definitions of DHD. Plante et al.^8^ explored the correlation between Cognitive Impairment (CI) and DHD, noting socioeconomic impacts and discharge destinations on hospital stays. CI often prolonged hospitalization, with prevalence varying from 4.6% to 63% among DHD patients. Future research should address CI's effects on healthcare systems and caregiver costs. Modas et al.^10^ evaluated tools for predicting prolonged hospitalization and DHD risk, focusing on cognitive function, age, daily living activities, mobility, and social support. Preventive methodologies and early discharge planning, coupled with tools like the multidisciplinary record, were effective in mitigating DHD. However, limitations included subjective assessments of social days and the need for more variables and diverse clinical testing. These studies highlight the complex nature of DHD and emphasize the need for comprehensive approaches that integrate individual and organizational interventions while addressing the challenges of defining and measuring its impact.

### 5.4 Topic 4: Transitional and social care and the role of funding

Multiple articles investigate global transitional and social care initiatives to improve patient discharge processes (Table 5). McGilton et al.^21^ focused on TCPs for older adults at the DHD risk, emphasizing interdisciplinary care teams and proactive admission. Spiers et al.^22^ explored the link between social care availability and healthcare utilization, concluding that more care home beds were associated with reduced DHD and healthcare spending. Limited focus on primary care, lack of home‐based care data, and absence of studies on affected population sub‐groups were noted. Mason et al.^25^ suggested that integrated funding may enhance care access but does not consistently yield significant health benefits or cost reductions. While earlier intervention and shorter stays were associated with mitigating DHD, challenges in implementing financial integration and linking information systems were outlined. Bhatia et al.^14^ highlighted the insufficiency of financial incentives in reducing DHD rates, advocating for interventions that allow local innovations in organizational and cross‐organizational approaches. These studies underscore the complexity of addressing DHD and advocate for comprehensive strategies integrating transitional and social care initiatives while acknowledging the challenges in implementing such interventions and the need for support policies to drive systemic changes.

### 5.5 Summary of topics synthesis and collating

Our review of systematic and scoping articles highlights DHD's multifaceted causes, ranging from service shortages to the complex needs of older adults. Although interventions like early discharge planning show promise, their efficacy remains uncertain due to research limitations. The emotional and physical toll on patients and caregivers underscores the need for improved communication and participatory strategies. Organizational resources and healthcare professionals' competencies are crucial in mitigating DHD, with nurse‐led interventions showing promise. Policy reform, enhanced communication, and targeted support for vulnerable groups are recommended. Table A1 summarizes the primary causes, recurring themes, interventions, and trends in DHD research.

## 6 DISCUSSION

This scoping review of systematic review articles delves into the multifaceted causes of DHD, including service shortages, hospital‐based delays, inadequate access to care, and patient‐specific factors like age and CI. Effective interventions, such as expanding community care services and implementing transition navigator roles, have been identified, though their efficacy is often questioned due to study limitations. The review emphasizes DHD's emotional and physical toll on patients and caregivers, stressing the need for timely information and policy revisions. Organizational and individual factors significantly influence DHD, with early discharge planning and care coordination showing promise. Despite efforts, research gaps persist, particularly regarding patient and caregiver experiences and intervention sustainability. Our review underscores the need for standardized, multidimensional approaches to address DHD effectively. Additionally, this review integrates findings from 23 articles spanning more than two decades, offering insights into prioritizing research areas and providing a framework for administrators and policymakers to enhance current practices sustainably.

### 6.1 Synthesis of systematic review articles findings

While we encountered various studies conducting systematic examinations, a unanimous definition and measurement standard for DHD was absent. Therefore, we propose a definition of the DHD phenomenon that takes into account key attributes from all review articles we studied as follows: DHD is a situation where a patient has recovered from their acute medical condition and is considered stable, no longer requiring the specialized resources and services provided in the hospital setting. However, due to nonmedical reasons, the patient is not able to transition to the next level of care and leave the hospital. The key attributes of this definition can be found in Table B1, item [1]. Additionally, several studies pointed to the importance of understanding patient characteristics, which is crucial for comprehending the root causes of this phenomenon.^3^, ^5^, ^6^, ^8^, ^10^, ^18^ The following discussion is the answer to our research questions.

What causes and factors contribute to ALC and DHD problems?

The DHD phenomenon and its contributing factors are complex and multifaceted, posing significant challenges to healthcare systems worldwide. Although defined by Micallef et al.,^5^ the term DHD lacks consistency across various studies, making it difficult to pinpoint the root causes and standardize interventions. The vagueness of terms such as ‘medically fit’ further complicates the understanding of DHD, as professional opinions on the matter can vary widely. This scoping review sought to elucidate the technical, administrative, and communicative issues that contribute to DHD and the role of healthcare management and leadership in addressing these issues. This review aims to address the DHD issue by exploring the inconsistencies in its interpretation, miscommunication and miscoordination, and the role of hospital management practices. The goal was to bridge the gap between research findings and practical applications and to highlight the need for a holistic and patient‐centric approach to mitigate DHD and improve healthcare outcomes.

### 6.2 Technical/administrative and standardized support

Although Micallef et al. have comprehensively defined the DHD term,^5^ several articles across all identified topics in this scoping review agreed that the lack of proper standardization makes it more challenging to outline the root causes and that even the term ‘medically fit’ is vague and can vary from one professional opinion to another.^3^, ^8^, ^13^, ^18^ Moreover, researchers have noted a concerning trend where studies fail to distinguish between DHD and inappropriate hospital use (patients who have been inappropriately admitted are being combined with those who have been appropriately admitted but are staying longer than necessary due to DHD), leading to conflated data. To ensure reliable outcomes and eliminate inconsistencies, healthcare professionals should embrace standardized definitions and protocols in their studies.^3^, ^8^, ^13^ On the other hand, numerous articles highlighted inadequate communication and coordination among hospital staff, patients, and healthcare and community institutions. This deficiency is a significant obstacle to minimizing DHD.^3^, ^5^, ^6^, ^13^, ^15^, ^16^, ^17^, ^18^, ^21^ This gap in research highlights the need for a discussion that is notably absent in the existing literature: understanding patients' viewpoints and, equally crucial, the caregivers' perspective. This aspect is central to addressing the communication challenges between healthcare professionals and patients.^6^, ^13^, ^23^, ^24^, ^31^ Additionally, the timing of discharges plays a significant role in DHD issues and subsequent readmissions. For instance, Lin et al.^9^ noted that night discharges led to increased mortality rates and premature discharges, emphasizing the impact of decisions made during night shifts on the prevalence of DHD.

### 6.3 Healthcare management and leadership

Many research findings agreed that the primary cause of the DHD issue lies in hospital management, internal processes, and inadequate coordination rather than insufficient capacity or patient cooperation.^3^, ^5^, ^9^, ^12^, ^15^, ^16^, ^17^, ^18^, ^20^, ^23^, ^24^ This finding contradicts the ‘More Beds, Better Care’ Act released by Health Ontario in 2022, which advocates for increased bed capacity as the solution sought by healthcare institutions, conflicting with existing research.^32^ Consequently, a noticeable gap between researchers and practitioners becomes apparent, emphasizing the need to advance and implement a practical, sustainable, and effective solution for DHD. Moreover, Åhlin et al.^12^ accentuated that previous attempts to bolster capacity have proven unsuccessful, indicating the necessity for more effective and innovative solutions to emerge.

What interventions and funding programs affected ALC and improved the cases of (DHD)?

### 6.4 Social/transitional/continuous care support and coordination

One study questioned the idea of solving DHD by building more transitional care facilities. Instead, it emphasized tackling root causes within hospital management.^21^ This viewpoint was confirmed by Cadel et al.,^6^ who argued that adding more transitional care while relieving pressure on ICU units would merely shift the problem from one sector to another.

### 6.5 Other interventions

Effective information sharing is pivotal in mitigating DHD, as highlighted in various studies.^6^, ^15^, ^16^, ^17^, ^18^, ^24^ It stands out as a successful intervention in addressing the issue. Information sharing encompasses collaboration among healthcare professionals, community‐based staff, patients, and caregivers. Hospitals that have embraced such collaborative efforts, integrating services like community‐based care programs, discharge planning, and patient information exchange, have experienced decreased readmissions and shorter hospital stays.^15^ Achieving effective information‐sharing between hospitals, community services, patients, and caregivers can be facilitated through several key strategies we discuss in Table B1, item [2].

### 6.6 Additional insight

Several articles highlighted the oversight of studying the impact of premature discharge, an aspect valuable for insights.^9^, ^15^, ^24^ Premature discharge, pressured by bed occupancy concerns, can lead to adverse outcomes, increasing readmissions and hospital stays. On the other hand, reviews indicate that when nurses manage the discharge or care transition, patients' process improves compared to physician‐led approaches.^9^, ^16^

## 7 PROPOSED FRAMEWORK FOR PROCESS IMPROVEMENT

Most articles state their recommendations in terms of focusing on improving the DHD problem. However, they should also explain how to implement their recommendations or suggested solutions in actionable steps. For over two decades, this problem has persisted without implementing long‐term solutions. This calls for research investigation and sustainable solutions that can be measured.^6^, ^25^, ^33^ Consequently, several articles have encouraged lean management and CPI to achieve long‐term results.^34^, ^35^, ^36^, ^37^ Therefore, our review aimed to guide healthcare policymakers, practitioners, and researchers in tackling the DHD problem.

**Figure 5:**
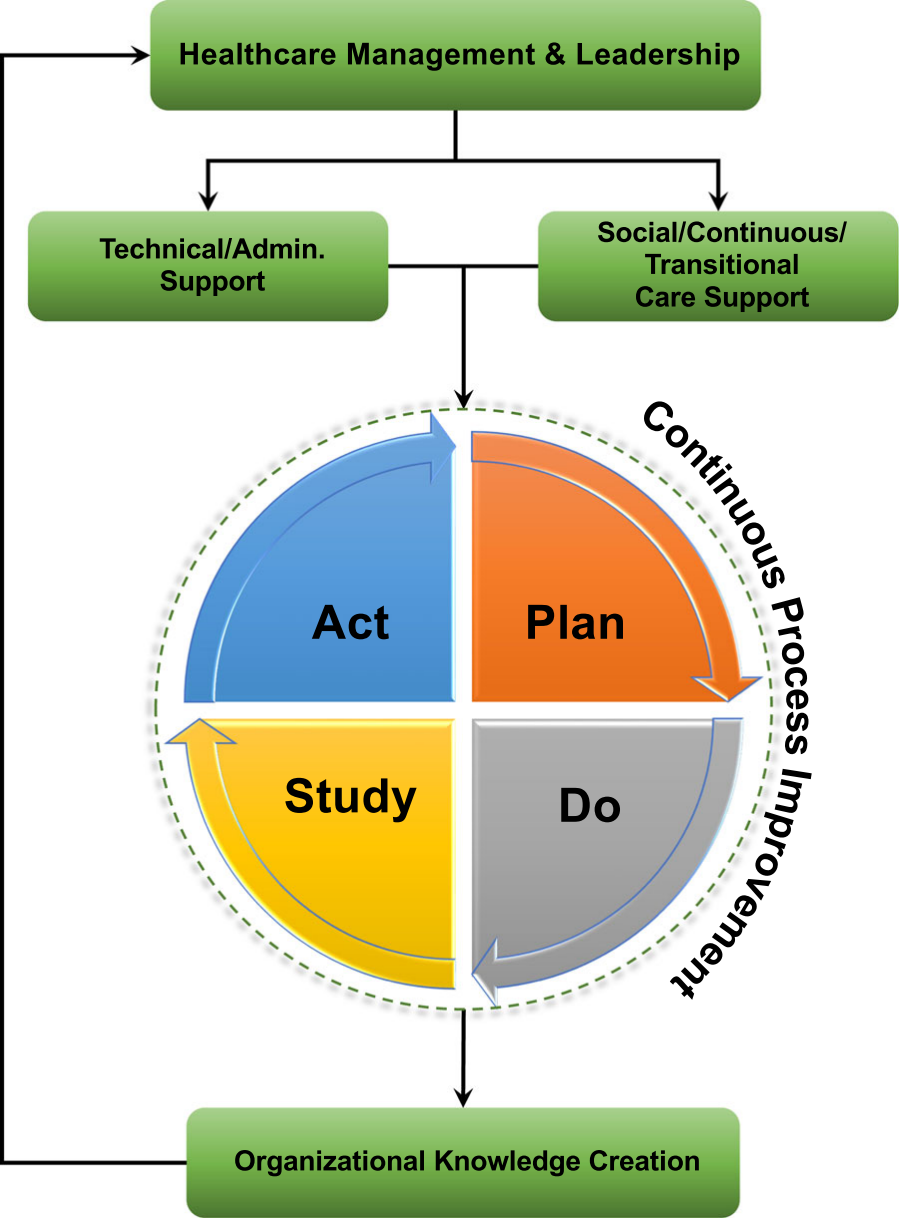
Healthcare process improvement framework to reduce Delayed Hospital Discharge.

From the synthesis of the causes, the main steps to improve the hospital process and management are through CPI modeling and review.^33^, ^34^, ^38^, ^39^, ^40^ CPI, also known as the Kaizen model, has been used in healthcare as a systematic and ongoing approach to enhancing healthcare management's quality of care, safety, and efficiency. It involves identifying, analyzing, and improving processes to optimize patient outcomes.^33^, ^41^ CPI is not a quick fix but a never‐ending journey that requires a commitment to ongoing investment and persistence.^33^, ^38^ While CPI has been used extensively in the industrial and manufacturing sectors, its effectiveness in healthcare is uncertain due to the healthcare systems' more complex and diverse nature.^34^, ^42^ However, CPI has shown promise in improving outcomes and reducing costs in various healthcare settings.^35^, ^36^, ^40^, ^42^ Figure 5 represents our scoping review framework, highlighting our contribution to Linderman's framework.^38^ Rooted in Linderman's process management improvement model, our framework incorporates adjustments from our review's discussions and recommendations. We introduce the CPI stage, a crucial addition for healthcare institutions seeking to establish a cohesive process improvement framework. Subsequent sections provide comprehensive insights into each component of our proposed framework.

### 7.1 Healthcare management and leadership

As explained in the discussion section, healthcare management is one of the main barriers to improving the status of DHD problems in hospitals worldwide. Making improvements to organizational systems requires commitment from senior management. Their involvement is essential to starting the process.^38^ This must consider both the locational context of the changes and a structure designed for the level of social and technical support that can be implemented.^14^, ^38^

### 7.2 Technical/administrative support

To guarantee systematic improvement, resources regarding information technology usage and healthcare personnel must be well structured and defined. Information technology can be used in several ways, including assessing the patient's physical and socioeconomic conditions. These factors have been shown to affect the expected length of hospital stay and the likelihood of discharge delays.^10^ In addition, hospitals should have a standard form of discharge that can be used when the patient is admitted and/or discharged to/from the hospital.^8^, ^10^, ^13^ It is imperative to train nurses on handling planned hospital discharges to ensure a seamless patient flow starting from the admission phase.^9^, ^16^

### 7.3 Social/transitional/continuous care support

**Figure 6:**
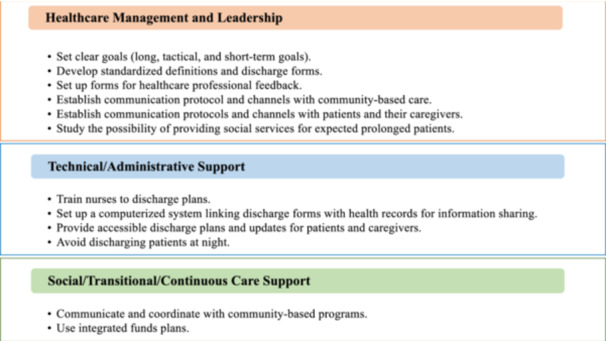
Actionable steps for continuous process improvement levels.

Social, transitional, and continuity of care are essential in improving DHD. It is important to share senior management's goals with the hospital team. This helps motivate them and achieve better performance outcomes. As discussed in the literature, it is also important to train and encourage the social and transitional team to avoid miscommunication^9^, ^18^, ^38^ (Figure 6).

### 7.4 Continuous process improvement

**Figure 7:**
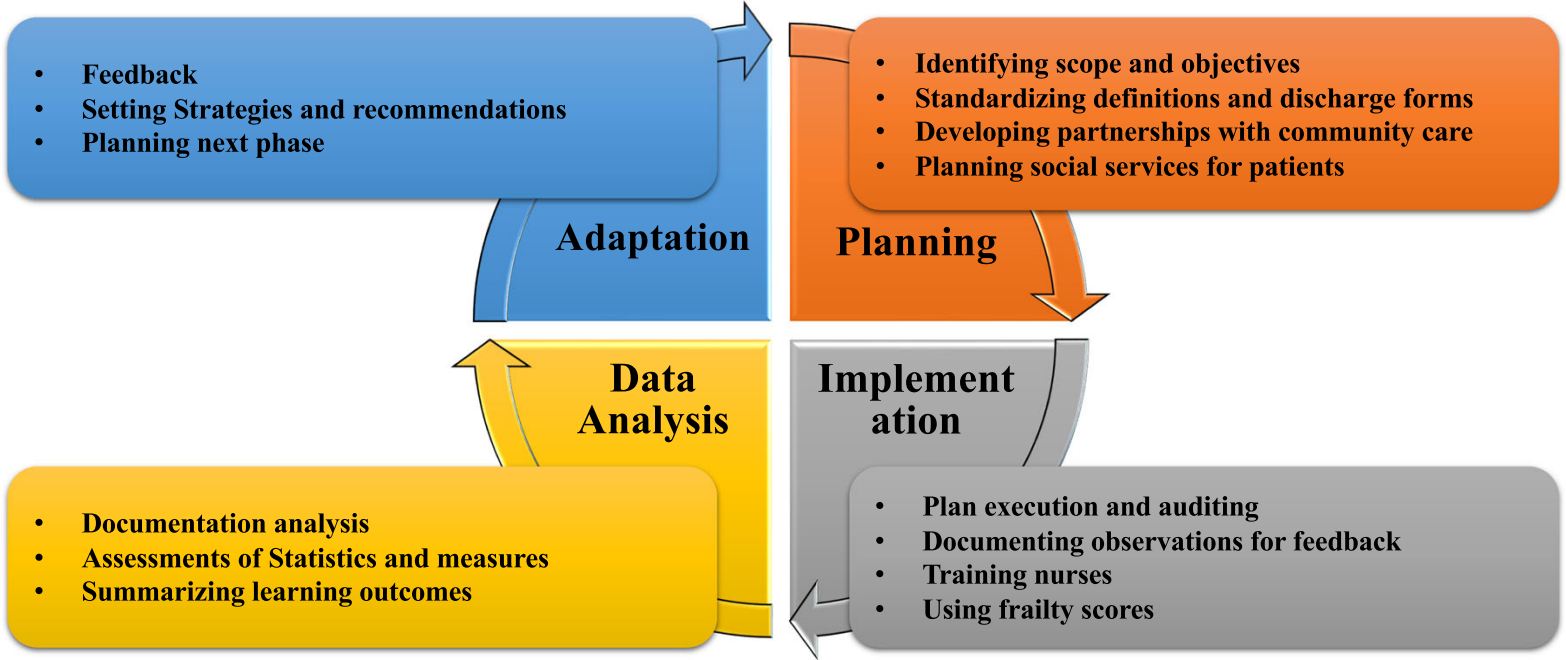
Continuous process improvement steps.

The framework's foundation, built upon Linderman's work, centres on process improvement techniques characterized by its four stages. These stages encompass planning, implementation, data analysis, and adaptation, fostering ongoing enhancement and alignment with evolving healthcare needs and insights. This approach draws inspiration from established methodologies such as the Plan‐Do‐Study‐Act (PDSA) cycle and promotes systemic, iterative healthcare delivery and outcomes improvements (see Figure 7)^34^, ^35^, ^40^, ^43^ (detailed explanation can be found in Table B1, item [3]):

1. Planning: Define objectives with direct input from patients and caregivers through focus groups or surveys. Develop a discharge plan with patient education, post‐discharge anticipation, and clear follow‐up expectations. Design patient‐friendly discharge forms with feedback sections.
2. Implementation: Clarify roles for patients and caregivers, provide training, and establish regular check‐ins to address issues.
3. Data Analysis: Assess patient satisfaction and effectiveness of caregiver support.
4. Adaptation: Based on feedback, revise materials or communication strategies. Continuously solicit feedback for ongoing improvement.

## 8 CONCLUSION

This scoping review thoroughly examines DHD by analyzing systematic reviews spanning over 24 years and encompassing more than 700 studies, providing a comprehensive overview. Our approach emphasizes the importance of incorporating patient and caregiver perspectives, highlighting the critical role of effective communication and management within healthcare environments. Through the scoping review, we emphasize the necessity for standardized definitions and sustainable discharge protocols, clarify debates surrounding discharge timing's impact on DHD, and assess the effectiveness of common solutions like increasing bed capacity. Rather than solely focusing on capacity expansion, we advocate for hospital management enhancements and interventions targeting foundational issues, such as social and transitional care support. This scoping review stands out by synthesizing findings from diverse literature on DHD and presenting a clear, actionable framework and action plan grounded in CPI principles. Building upon Linderman's 2010 work, we propose a unique CPI model tailored to the complexities of healthcare aimed at guiding healthcare managers and policymakers in effectively addressing DHD. Furthermore, we provide a comprehensive yet concise definition of DHD and elucidate the key attributes that could underpin this definition. While the review is thorough, we acknowledge the limitation of not including practitioner consultations, an aspect we plan to explore in future research endeavors.

## AUTHOR CONTRIBUTIONS

**Alyaa Abdelhalim**: Conceptualization; investigation; writing—original draft; methodology; visualization; writing—review and editing; software; formal analysis; data curation; resources. **Manaf Zargoush**: Writing—review and editing; supervision; validation; conceptualization; visualization; project administration. **Norm Archer**: Writing—review and editing; supervision; validation. **Mehrdad Roham**: Writing—review and editing; supervision; validation.

## CONFLICT OF INTEREST STATEMENT

The authors declare no conflict of interest.

## Data Availability

Data sharing is not applicable to this article as no datasets were generated or analysed during the current study.

## APPENDIX A ANALYSIS OF CAUSES, BARRIERS, AND INTERVENTIONS FOR DELAYED HOSPITAL DISCHARGES

Table A1

**Table A1:** Analysis of causes, barriers, and interventions for delayed hospital discharges.

[1] The main causes and barriers contributing to delayed hospital discharges, as outlined in this review, are multifaceted and include:	1. Lack of rehabilitation services: Patients often face delays due to the unavailability of appropriate rehabilitation services that are necessary for their continued recovery after hospital discharge. 2. Hospital‐based delays: Inefficiencies within the hospital system, such as bed shortages or administrative hold‐ups, contribute to delayed discharges. 3. Awaiting transfer: Patients sometimes have to wait for transfer to other hospitals or care facilities, which can cause significant delays. 4. Faulty organizational management: Inadequate discharge planning and transfer of care problems, often due to poor organizational management, lead to extended hospital stays. 5. Age‐related factors: Older, frail, and dependent individuals are particularly vulnerable to delayed discharges due to their complex health needs. 6. Inadequate access to care: Barriers such as inadequate access to residential care, domiciliary support, and limited hospital capacity prevent timely discharge. 7. Poor communication: Communication deficits between hospitals, community health and social care providers, and within hospital departments hinder the discharge process. 8. Funding difficulties: Financial constraints and funding issues can delay the provision of necessary post‐discharge services. 9. Patient and carer‐related factors: The specific needs of older people with mental health problems and those from minority ethnic communities are not always adequately addressed, leading to delays. 10. Delayed assessment and coordination: Delays in patient assessment and lack of coordination between healthcare providers contribute to extended hospital stays. 11. Cognitive impairment: Patients with cognitive impairments often experience longer hospital stays due to the need for specialized care and suitable discharge destinations. 12. Socioeconomic factors: Patients’ socioeconomic status can impact the availability and coordination of post‐discharge care, leading to delays. 13. Policy struggles: Common policy issues across different countries, such as the lack of jurisdiction‐wide approaches, exacerbate the problem of delayed discharges.
[2] The causes or barriers to achieving timely discharge that were repeated in more than one study across topics include:	1. Lack of rehabilitation services: Multiple studies highlighted the absence of adequate rehabilitation services as a barrier to timely discharge. 2. Hospital‐based delays: Several articles identified hospital‐based delays, such as long lead times and inefficient patient transfer processes, as significant contributors to DHD. 3. Awaiting transfer: The need for patients to await transfer to other hospitals or care facilities was mentioned as a cause for delayed discharges. 4. Inadequate access to care: Inadequate access to residential care, rehabilitation services, domiciliary support, and limited hospital capacity were repeatedly cited as barriers. 5. Poor communication and coordination: Communication deficits and inadequate coordination of care, including poor clinical handover practices, were commonly reported as causes of DHD. 6. Funding difficulties: Financial constraints and difficulties in implementing financial integration were noted as barriers to timely discharge. 7. Resource availability: A recurring theme was a shortage of ICU beds and other hospital resources, leading to delayed discharges. 8. Inadequate discharge planning: Faulty organizational management and inadequate discharge planning were identified as root causes of DHD. 9. Age‐related factors: The challenges associated with the discharge of older, frail, and dependent individuals were frequently mentioned. 10. Cognitive impairment (CI): The association between CI and increased length of stay or DHD was highlighted in multiple studies.
[3] The review identifies several interventions that have been effective in mitigating the problem of delayed hospital discharges:	1. Structured discharge plans: Tailoring discharge plans to individual patient needs has been shown to reduce hospital length of stay and unscheduled readmissions. 2. Timely discharge planning: Initiating discharge planning early during hospital admission helps organize post‐discharge services and can reduce hospital length of stay. 3. Care coordination: Coordinating care among different healthcare providers and services has demonstrated positive impacts on reducing hospital admissions, readmissions, and length of stay, particularly for frail older individuals. 4. Preventive health checks and geriatric assessment units: These interventions focus on the specific needs of older patients and have been effective in reducing hospital stays. 5. Care home liaison and community‐based rehabilitation services: These services help bridge the gap between hospital care and community or residential care, aiding in the transition and reducing readmissions. 6. Nurse‐led discharge planning: Involving nurses in the discharge planning process has helped mitigate delayed discharges by ensuring that patient care continues seamlessly after leaving the hospital. 7. Development of discharge facilitation Tools: Tools such as ‘discharge before noon’ policies and tracking mechanisms help identify and address factors hindering early discharge. 8. Involvement of general practitioners and Social Care Staff: Engaging these professionals in the discharge process ensures better coordination and continuity of care. 9. ICU Liaison and outreach services: These services have been found to improve the flow and performance of ICUs, resulting in shortened hospital stays and decreased patient mortality. 10. Legislative measures: For example, the Community Care (Delayed Discharges etc.) Act 2003 in England, which charges social services departments for hospital beds occupied by patients awaiting social services provision, has influenced discharge processes. 11. Multidisciplinary care and case management: These approaches involve various healthcare professionals working together to manage patient care, which can reduce hospital length of stay. 12. Telehealth services: The use of telehealth can facilitate post‐discharge follow‐up and care management, potentially reducing the need for readmission. 13. Early discharge planning: Planning for discharge at the beginning of the hospital stay has been associated with fewer hospital days for acutely ill or injured patients.

Abbreviation: DHD, Delayed Hospital Discharge.

## APPENDIX B PROPOSED FRAMEWORK, DHD DEFINITION AND CPI

Table B1

**Table B1:** Proposed DHD definition, framework, and CPI detailed steps.

[1] Key Attributes of the proposed DHD definition in Section 6.1. Synthesis of Systematic Review Articles Findings	1. Medical readiness: The patient has resolved the acute medical condition requiring hospitalization and is clinically stable (medically fit), with no need for the specialized resources or services of the acute care setting. 2. Nonmedical barriers: The delay is attributed to factors beyond the patient's medical condition, such as incomplete assessments, awaiting placement in a more suitable care setting (e.g., rehabilitation, long‐term care facility, or home care), or coordination challenges among healthcare providers and services. 3. Systemic implications: The delay impacts hospital throughput, leading to inefficient use of healthcare resources, increased risk of iatrogenic harm to the patient, and potential exacerbation of health conditions due to extended hospital stays. 4. Patient‐centric perspective: Recognizing the patient's experience and the psychosocial impact of delayed discharge, including the risk of functional decline, social isolation, and psychological distress. 5. Healthcare coordination: Emphasizes the need for effective communication and collaboration within hospital systems and between acute care and community or social care services to facilitate timely and safe patient transitions. 6. Resource utilization: Addresses the broader implications for healthcare systems, such as increased costs, bed shortages, and the cancellation of elective procedures due to prolonged occupancy of acute care beds by patients ready for discharge.
[2] Key strategies for achieving effective information sharing between hospitals, community services, patients, and caregivers:	1. Cross‐sectoral access to information: We underscore the importance of electronic access to information across organizational and health sector boundaries. This can be facilitated by implementing integrated health information systems allowing real‐time patient data sharing. Such systems ensure that community care providers can access up‐to‐date medical records, discharge plans, and follow‐up care requirements. 2. Facilitating partnerships: We recommend establishing networks of care with shared accountability and communication efforts. This involves collaboration between different healthcare providers and organizations to ensure patient transition. Regular meetings and collaborative platforms can help maintain open lines of communication between hospitals, primary care, social services, and other community care organizations. 3. Tools and guidelines: The development and implementation of standardized communication tools and guidelines are essential for streamlining the discharge process. These tools may include discharge summaries, checklists, and protocols that ensure all necessary information is communicated clearly and promptly to community service providers. 4. Transdisciplinary approach: The inclusion of interdisciplinary communication meetings to discuss bed availability, discharge planning, and coordination of care. 5. Discharge coordinator or liaison nurse: The involvement of a dedicated professional, such as a discharge coordinator or liaison nurse, can facilitate the assessment of patients for transfer, coordinate transfers, and liaise with community care staff during discharge. This role can bridge the gap between hospital and community services, ensuring that information is effectively communicated. 6. Patient and caregiver engagement: We emphasize the need for improved communication and enhanced patient engagement. This can be achieved by involving patients and caregivers in the discharge planning process, ensuring they understand the care plan and are prepared for the transition from hospital to home or another care setting.
[3] CPI Steps	1. Planning: In this initial stage, healthcare professionals should define the objectives informed by direct input from patients and caregivers. Engage them in focus groups or surveys to understand their needs and expectations. Develop a discharge planning process that includes patient education, anticipates post‐discharge needs, and sets clear expectations for follow‐up care. Standardized discharge forms should be designed to be patient‐friendly and include sections for patient feedback and concerns. 2. Implementation: As changes are executed, ensure that the patients’ and caregivers’ roles are clearly defined and supported. Provide training for nurses responsible for discharge planning and training for patients and caregivers on post‐discharge care, medication management, and warning signs of potential complications. Implement a system for regular check‐ins with patients and caregivers to address any issues during the discharge process. Document all patient and caregiver interactions to identify areas for improvement. 3. Data analysis: Analyse data from healthcare team observations and patient and caregiver feedback. Assess patient satisfaction with the discharge process and the clarity of the information provided. Evaluate the effectiveness of the support and education provided to caregivers. Use this data to understand where the discharge process meets patient needs and where it falls short. 4. Adaptation: Based on the analysis, adapt the discharge process to better meet the needs of patients and caregivers and align with the healthcare professional feedback and observations. This could involve revising educational materials, improving communication strategies, or altering follow‐up procedures. Ensure that any changes are communicated back to patients and caregivers and that their ongoing feedback is solicited to continue the improvement cycle.

## APPENDIX C COMPREHENSIVE CHARACTERISTICS OF ALL THE ARTICLES INCLUDED IN THE SCOPING REVIEW

Table C1

**Table C1:** Characteristics of all the articles.

References	Articles reviewed	Interventions (if included)	Reasons for DHD	Other improvement factors	Key Findings
Philp et al.^15^	48	1. Care coordination 2. Preventive health checks 3. Care home liaison in the prevention of admission to hospital 4. Geriatric assessment units and orthogeriatric units targeting frail older people. 5. Discharge planning 6. Information sharing and providing rehabilitation services.	Not mentioned	Not mentioned	No evidence of impact on hospital bed use from interventions such as multifactorial fall prevention services, day hospital services, medication reviews, exercise programs in the community, nutritional enhancement in hospitals, and nurse‐led transitional care units.
Luis et al.^29^	13	1. The implementation of multiple‐component interventions linked to discharge planning has been identified as a potential strategy to reduce inefficiency and minimize unnecessary stays. 2. A multidisciplinary quality improvement initiative was mentioned as an intervention to identify discharge barriers in general medicine patients. 3. The ongoing expansion of care to dependent people, as mandated by the ‘Law to promote personal autonomy and care for dependent people’, has been shown to help reduce inefficiencies in hospital care. 4. Interventions aimed at reducing inappropriate admissions and stays have been mentioned. 5. Some interventions involved analyzing the hospital costs saved by reducing inadequate stays.	1. Multifactorial Issues influenced by factors internal or external to hospitals, as well as personal factors. 2. The medical characteristics of the patients, particularly the elderly, frail, and dependent individuals, were identified as significant contributors to delayed discharge. 3. Family refusal of home‐based care and lack of alternative care centres accounted for a significant percentage of delayed discharge cases. 4. The complexity of patients’ processes was linked to delayed discharges. 5. Certain medical conditions, such as acute cerebrovascular disease, were associated with a higher prevalence of delayed discharges. 6. Inadequate social care and the inability to balance the care needed by the patient and work life were identified as reasons for delayed discharge.	NA	1. The review highlighted the prevalence and average duration of delayed discharge, as well as the potential negative repercussions on patients’ health and well‐being, including increased risk of healthcare‐related infections, functional impairment, and negative emotional impacts. 2. The study also identified the limited literature available on delayed discharge in Spain, emphasizing the need for further research, particularly focusing on socio‐familial characteristics and the perspective of patients and families.
Åhlin et al.^12^	92	NA	1. Long lead times 2. Inefficient capacity coordination 3. Inefficient capacity 4. Large capacity utilization variation 5. Inefficient capacity utilization 6. High work in the process 7. Inefficient patient process transfer 8. Inefficient support process transfer 9. Unpredictable inflow variation 10. Changing demand 11. Inefficient outflow process 12. Low interorganizational coordination	1. Lack of staff 2. Lack of standards and routines 3. Insufficient operational planning 4. Lack of IT functions	1. Several root causes are more easily addressed and can lead to capacity improvements without increasing expenditures. 2. Recommended to use of improvement agents and healthcare managers at hospitals
Goncalves‐Bradley et al.^19^	19	Discharge Planning	NA	1. A small reduction in hospital length of stay for those allocated to discharge planning in trials recruiting older people following a medical admission 2. A lower readmission rate in the discharge planning group at three months of discharge 3. Uncertain whether discharge planning has an effect on days in the hospital due to unscheduled readmission for patients with a medical condition 4. Discharge planning may not affect the proportion of patients discharged to home rather than to residential care 5. Discharge planning may lead to increased satisfaction for patients and healthcare professionals	1. A structured discharge plan tailored to the individual probably brings about a small reduction in hospital length of stay and unscheduled readmission for elderly patients with a medical condition. 2. Educating patients in the treatment group about medication and side effects might have made them more likely to visit the emergency department
Tipton et al.^30^	19	1. Discharge planning 2. Geriatric assessment or consultation 3. Medication management 4. Clinical pathways 5. Inter‐ or multidisciplinary care 6. Case management 7. Hospitalist services 8. Telehealth	1. Healthcare Disparities. 2. Medically Complex Needs. 3. Environmental Factors: including unique resources, personnel, leadership, and infrastructure specific to each setting. 4. Admission Process and Discharge Disposition. 5. Structural Interventions. 6. Social and Economic Barriers: The lack of research focused on patients who face severe social or economic barriers to achieving and maintaining wellness before, during, and after hospitalization contributes to the challenges in addressing delayed discharge.	NA	1. The report highlights that rigorous systematic reviews have not consistently demonstrated the effectiveness of strategies to reduce length of stay (LOS) for medically complex, high‐risk, or vulnerable patients. 2. The report identifies research gaps, including the lack of detail regarding hospital settings and implementation context, the reliance on studies conducted outside the United States, and the need for additional funding for primary research. 3. Health system leaders, researchers, and policymakers are urged to collaborate to develop informed strategies for LOS reduction.
Glasby et al.^16^	21	NA	1. Lack of rehabilitation 2. Hospital‐based delays 3. Awaiting transfer to another hospital/wider NHS services 4. Assessment delays/uncertainty over aftercare 5. Funding difficulties/delays 6. Awaiting care home placement/availability 7. Awaiting domiciliary/community package/lack of community services 8. Staff shortages (various professions/agencies) 9. Housing/aids and adaptations/social 10‐ circumstances user/carer‐related factors	NA	1. Different definitions of delayed discharge raise concerns about the accuracy of official statistics. 2. The literature lacks a patient and carer perspective.
Goncalves‐Bradley et al.^19^	19	Discharge planning	Not mentioned	1‐ Discharge planning resulted in a small reduction in hospital length of stay and a lower readmission rate. 2‐ Discharge planning may lead to increased satisfaction for patients and healthcare professionals	1. Educating patients in the treatment group about medication and side effects might have made them more likely to visit the emergency department
van Sluisveld et al.^16^	11	1. Implement liaison nurses to improve the communication and coordination of care between ICU and ward healthcare professionals. 2. Handover forms to facilitate the timely handover from ICU to ward healthcare professionals.	1. Poor information transfer is a common patient safety issue in all types of handover settings 2. The use of an inappropriate intervention about the underlying healthcare problem 3. Measurement of inappropriate outcomes 4. Suboptimal research population, such as a low mortality rate at the baseline	NA	1. Using an ICU discharge form is an effective intervention. 2. Effective handover is a timely transfer with complete information on the transferred patient.
Rameli et al.^17^	27	NA	1. Frailty 2. Multimorbidity 3. Lack of information sharing with patients and caregivers 4. Medication errors 5. Lack of community coordination with general practitioners 6. Lack of follow‐up	Measures: 1. Functional outcomes, including frailty 2. Quality of Life 3. Economic factors 4. Patient‐centred factors 5. Medication management 6. Caregiver outcomes 7. Community services link‐in 8. Follow‐up and readmission.	1. There were no specific follow‐up studies on outcomes after the intervention. 2. Effective communication between hospital and primary care teams enables continuity of care post‐discharge. 3. Greater emphasis must be placed on frailty scoring to guide the discharge destination.
Landeiro et al.^3^	64	NA	1. At an individual level, the specific needs of certain patient groups remain unaddressed. 2. At an organizational level, delays in providing acute hospital services impact delayed discharges. 3. At a structural level, poor communication between health care and social care.	Not mentioned	1. Local factors play an important role in reducing delayed discharges. 2. The need for internationally recognized parameters to determine when a patient is medically ready for discharge 3. Other factors may be associated with the proportion of delayed discharges, such as the level of social support of the patients, level of income, level of dependency on daily living activities, and comorbidities.
Cadel et al.^6^	66	1. Information Sharing 2. Tools and guidelines 3. Practice changes 4. Infrastructure and Finance 5. Other initiatives	Not mentioned	Not mentioned	1. Adding more intermediate care beds may alleviate pressures in acute care in the short term. 2. Place an equal focus on optimizing patient and caregiver experiences and outcomes. 3. Lack of information and details on the implementation strategy, setting, and population characteristics. 4. Lack of consistency in how delayed discharge was defined.
Micallef et al.^5^	64	NA	1. Conflicts of interest between health professionals. 2. procedural delays. 3. Confusion and redundancy in the care plan. 4. Lack of discharge planning and poor communication 5. Insufficient statutory services.	1. The rate of delayed discharges is due to hospital organization and management issues.	Delayed discharges cause: 1. Severe accident and and emergency. 2. Overcrowding and bed‐blocking. 3. inevitable negative financial implications.
Bhatia et al.^14^	6	Policy Programs	Not mentioned	NA	1. A single approach may be insufficient to tackle the delayed discharge/ALC challenge. 2. Attempts to reduce delayed discharge/ALC rates should be multicomponent, tailored to the local context. 3. Financial incentives alone are insufficient to reduce delayed discharge/ALC rates.

Table C2

**Table C2:** Full description of the aim of the study, inclusion, and exclusion criteria of each selected article.

References	Aim of study	Inclusion criteria	Exclusion criteria
Philp et al.^15^	To help improve our understanding of the interventions that appear to work and those that have been less successful in reducing hospital bed use.	Studies from 2007 to 2013	1. Those that did not include older people or frail older people as the main target population 2. Studies that only included older people with specific long‐term conditions 3. Studies of lower quality, for example, single case studies, audit studies 4. Those that did not include a comparison population.
Åhlin et al.^12^	1. To explore existing research on what factors prevent swift and even patient throughput at hospitals. 2. To synthesize those factors into themes, main barriers, and underlying root causes.	1. Contain an abstract; 2. Be written in English; 3. Be a qualitative or quantitative, empirical primary study on patients receiving hospital care; 4. Contain at least one description of a patient process‐related barrier; 5. Have been published between 1st January 2010 and 1st November 2020	1. Primary care or care within a rehabilitation setting; 2. Healthcare processes not relating to the hospital patient process; 3. Description of theories, methods, or models without empirical data; 4. Editorials or policy statements without immediate empirical support; 5. Literature reviews
Glasby et al.^16^	Review the UK literature on delayed hospital discharges and older people to identify and explore the rate and causes of delayed hospital discharges, together with policies and practices that may reduce delayed discharges and improve the experiences of older people.	1. Relating to delayed hospital discharge and older people. 2. Focused on documents exploring the rate and causes of delayed hospital discharge for older people in the United Kingdom.	1. Material published or based on data collected before 1993 2. Local inspections where findings are summarized in a national report 3. Articles reporting findings from studies already included in the review 4. Discharge from non‐acute care or accident and emergency departments, 5. The discharge of people aged under 65 (unless a significant proportion of the sample was older people) 6. Historical estimates of the rates of delayed discharge in official reports, where there is a more up‐to‐date figure.
Peltonen et al.^18^	1. To explore the incidence of patient admission and discharge delays in critical care and to identify organizational factors associated with these delays. 2. Critically analyse current literature on factors influencing the ICU patient discharge process. 3. To focus on areas that may be amenable to change but are not as frequently considered.	1. Either qualitative or quantitative studies; 2. Carried out in any ICU/HDU; 3. Directly related to the ICU patient discharge process. 4. The included studies were original research with 5. No restriction on design. 6. The type of participants included adult patients in intensive care, critical care, and high dependency. 7. The studies focused on organizational 8. factors (i.e., health service research) connected with patients’ admission and discharge delays in critical care. 9. The types of outcomes were patient admission, transfer, and discharge delays.	1. If they were not specifically about ICU/HDU patient discharge. 2. The outcomes were not about the admission or discharge process. 3. The allocation was not about critical care. 4. The focus was not on decision‐making concerning care coordination. 5. The participants were children or neonates.
Mason et al.^25^	Whether integrated funds have been realized in practice by facilitating coordinated care, and whether this could support improvements in patient experience and health and social care outcomes, reduce avoidable hospital admissions and delayed discharges, and so reduce costs.	1. Case studies/reviews of schemes integrating financial or resource flows across health and social care a. With or without evaluations/evidence/theoretical analysis b. Services for adults 2. Mechanisms for allowing resources to follow patients between health and social care organizations 3. Published in or after 1999 4. English language	1. Reports of systems from low‐income countries 2. Clinician/dentists/patient payment reimbursement mechanisms 3. Personalized budgets 4. Integrated systems for children's services 5. Financial integration across different healthcare settings only (not including social care) 6. Financial integration across different social care settings only (not including health care) 7. Articles with insufficient detail to judge inclusion criteria 8. Commentary, opinion pieces, and descriptive articles that provided no relevant empirical evidence
Spiers et al.^22^	To systematically review evidence on the relationship between the availability and supply of social care and healthcare utilization (HCU) for older adults.	1. Older adults aged 60+. 2. Any service for those aged 60+ that aims to enable independence and/or assist with activities of daily living. 3. Studies investigating associations and/or predictions between variations in social care accessibility and health care utilization. 4. Any language. 5. Studies from the OECD classification of high‐income countries. 6. Studies from 2000 ‐ 2019. 7. Journal articles, grey literature.	1. Young adults. 2. Studies which do not look at some form of variation in social care. 3. Studies of interventions for social care users that are designed to influence health care use 4. Books and conference abstracts where there is no corresponding full‐text publication.
Goncalves‐Bradley et al.^19^	Does discharge planning improve the appropriate use of acute care? 1. Effect of discharge planning on length of stay in hospital compared to usual care. 2. Effect of discharge planning on unscheduled readmission rates compared to usual care. 3. Effect of discharge planning on unscheduled readmission rates compared to usual care.	The trials included discharge planning interventions, which included assessment, planning, implementation, and monitoring phases.	1. Due to multifaceted interventions, of which discharge planning was only a minor part. 2. Trials that did not include an assessment phase.
van Sluisveld et al.^16^	To systematically review interventions with the aim of improving the quality of patient handover between ICU and general ward healthcare professionals at ICU discharge and to evaluate the overall effects of these interventions.	1. Inclusion of patients or healthcare professionals involved in the handover from the ICU to a step‐down unit or ward. 2. Inclusion of an intervention explicitly describing one or more components that aimed to improve the handover of care between healthcare professionals from the ICU and those of a step‐down unit or general ward. 3. The study design was experimental or quasi‐experimental, such as a (cluster) randomized controlled trial, cohort study, or a noncontrolled before ‘after study’. 4. There was at least one process or outcome measure addressing the quality or safety of the discharge.	Studies not available in full‐text format were excluded.
Lin et al.^9^	Critically analyse current literature on factors that influence the ICU patient discharge process. It examined how organizational factors, individual factors, and teamwork factors influence ICU patient discharge	1. Either qualitative or quantitative studies 2. Carried out in any ICU/HDU 3. Directly related to the ICU patient discharge process.	Articles were excluded if they were not about ICU/HDU patient discharge.
Modas^10^	A quick identification at the time of admission of patients at risk of discharge problems.	1. Studies that address evaluation instruments for identifying patients at risk of prolonged hospitalization time 2. Comprising patients of all age groups hospitalized in acute and/or chronic situations in different medical specialties. 3. Primary studies and reviews, 4. Qualitative and quantitative studies, with abstract and full text available, 5. Written in Portuguese, Spanish, and English languages. 6. No temporal or geographical limit was defined to obtain the maximum of existing evidence.	Not mentioned
Rameli et al.^17^	To summarize the current literature on: 1. Discharge pathways or initiatives in place for CDP. 2. Patient outcomes associated with CDP across nine domains.	1. Patients aged 65 years and older in the acute hospital setting. 2. Delayed discharge from the acute hospital setting to home or intermediate care 3. Peer‐reviewed articles focusing on CDP, complex discharge units, LOS, or the impact of delayed discharge 4. published between January 2001 and December 2021 5. Discharge strategies to improve patient outcomes.	1. Patients aged below 65 years 2. Articles not published in the English language 3. Patients admitted for elective cardiology interventions or surgical or vascular or orthopedic procedures or patients who were post‐stroke and receiving rehabilitation 4. Patients admitted under psychiatry or oncology services 5. Patients who escalated to the intensive care unit (ICU) with a protracted ICU course and who later died.
Landeiro et al.^3^	To examine: 1. The proportions of delayed discharges of older peopleworldwide, including synthesizing the results of publishedstudies on the proportions of delayed discharges to identify the main factors associated with them. 2. The economic impact of delayed discharges on health	1. Study populations were inpatients in acute hospitals. 2. The mean age of the sample population was 65 years or older. 3. Data were reported on the number of days of delayed discharges or the proportions of delayed discharges. 4. Studies were comparative (randomized controlled trials and nonrandomized studies) or observational studies (cross‐sectional studies, cohort studies, case ‘control studies’, and database studies). 5. Journal articles and theses were included.	not mentioned
Cadel et al.^6^	1. To review what delayed discharge initiatives entail (e.g., characteristics, outcomes) 2. to identify gaps in the literature to inform future studies.	1. Focused on delayed discharge 2. Included an initiative to address delayed discharge 3. Involved a hospital setting 4. Published between 1 January 2004 and 16 August 2019 5. Peer‐reviewed or grey literature.	1. Focused on changing the threshold/timing of discharge (early discharge) 2. Books, book chapters, opinion pieces, or editorials 3. Grey literature that did not sufficiently describe the initiative implemented (e.g., implementation process, location, population, impact); 4. Protocols, trial papers, or chart reviews 5. Conference abstracts or articles without an accessible full‐text
Everall et al.^23^	To summarize the literature on patient and caregiver experiences with delayed hospital discharge	1. Patient or caregiver population 18 years or older 2. Delayed discharge (i.e., medically cleared with no suitable next destination available) from a hospital setting 3. Included experiences with delayed discharge 4. Peer‐reviewed or grey literature 5. Published between 1 January 1998 and 16 July 2018	1. Were a book, book chapter, editorial, opinion piece, study protocol, case law or trial report, or abstract with no full‐text articles 2. Focused only on the length of stay, impacts of delayed Discharge on the hospital system, or patient health outcomes (excluding experience) 3. Only described indicators/determinants of delayed discharge. 4. Scoping and systematic reviews
McGilton et al.^21^	To answer: 1. What are the socio‐demographic and/or clinical characteristics of older patients served by TCPs? 2. What do TCPs provide the core components? 3. What patient, caregiver, and health system outcomes have been investigated, and what changes in these outcomes have been reported for TCPs?	1. If they reported models of TCPs that served community‐dwelling older adults (aged 65 years or older) experiencing or at risk for delayed discharges 2. if they reported examining processes and/or outcomes used to evaluate the programs.	Studies that focused on standards of care in transitioning from acute care to home (e.g., rehabilitation post fracture or stroke) or involved home‐based TCPs
Rojas‐Garcia et al.^24^	To systematically review experiences of a delay from the perspectives of patients, health professionals, and hospitals and its impact on patients’ outcomes and costs.	1. Quantitative data on the impact of delayed discharge on health outcomes. 2. Qualitative data on experiences of a delay from the perspectives of patients, health professionals, and hospitals. 3. Information on costs of delay due to unnecessary bed days. 4. Only studies written in English, published since 2000, and conducted in the OECD	1. Research focusing on mental health, maternal and child and adolescent health, and palliative care. 2. Abstracts, editorials, commentaries, and book reviews were excluded because the review focused on primary research.
Plante et al.^8^	To identify evidence on the impact of cognitive impairment (CI) on acute care hospital LOS/DD to make precise regionally‐based recommendations for reducing LOS or DD in cognitively impaired patients	1. Participants are In‐patients 45 years old 2. Intervention/exposure in Acute care hospitalization 3. Comparison based on the Cognitive status 4. Outcomes measured are LOS or DD 5. All types of study design	1. Book, book chapters, editorials, commentaries, and letters 2. Studies on in‐patients with intellectual disabilities at the end of life or in palliative care 3. Setting other than an acute care hospital 4. Incomplete data, such as a conference abstract
Micallef et al.^5^	1. To investigate the conceptual and operational definitions of delayed discharges 2. Explore the causes and effects of delayed discharges 3. Identify interventions to decrease the impact of delayed discharges in acute healthcare settings.	1. Articles written in the English language 2. Research articles published from 1990–2019 3. Only research conducted in acute hospital settings was included 4. Solely adult ward settings were included 5. Only primary evidence‐based research articles were included 6. Articles taken from an organizational perspective	1. Articles written in other languages 2. Articles published pre‐1990 and post‐2019 3. Studies conducted in other areas were excluded 4. Paediatric ward settings were excluded 5. Opinion articles and other speculative write‐ups were 6. excluded 7. Articles taken from patients’ perspective
Bhatia et al.^14^	to identify existing initiatives aimed at reducing delayed discharge from the hospital, ALC days, or length‐of‐stay	Not mentioned	1. Focus exclusively on Canada. 2. Not original, primary, or peer‐reviewed sources. 3. The focus of the intervention was not to reduce DHD. 4. The intervention involved discharge to other care settings. 5. The intervention involved discharge from other settings. 6. The study design was not evaluative. 7. The study used a quality‐improvement methodology. 8. The intervention did not reduce DHD. 9. The study involved a single unit. 10. The full text of the source was not accessible
Fox et al.^20^	To compare the effectiveness of early discharge planning to usual care primarily in reducing index length of hospital stay, hospital readmissions, and readmission length of hospital stay and secondarily in reducing mortality and increasing satisfaction with discharge planning and quality of life for older adults admitted to hospital with an acute illness or injury.	1. Published and unpublished randomized control and quasi‐experimental trials with parallel controls that compared early discharge planning to usual care for adults aged 65 and older in the acute illness or injury phase. 2. Include at least one primary outcome (hospital readmissions) or secondary outcome (mortality).	1. Those that were unavailable in English or French. 2. Compared usual care units to acute care for elders units, which provided early discharge planning as one of two or more ACE intervention components. 3. Compare usual care to exercise programs. 4. Include historical control groups. 5. Included patients in the sub‐acute or post‐acute phase. 6. Included social admissions or receiving palliative care.
Luis et al.^29^	This study aims to analyse delayed effective discharges for nonmedical reasons for patients admitted to acute care hospitals in Spain.	1. Quantitative data on the impact of delayed discharge on health outcomes (e.g., care quality, satisfaction, number of infections, mental health, mortality, morbidity, readmissions, and functionality). 2. Qualitative data on delayed discharge experiences from the perspective of patients (e.g., perceived impact on health or patients experience), health professionals, and hospitals. 3. Information on delayed discharge costs due to unnecessary hospitalization days.	1. Summaries, editorials, comments and book reviews were excluded.
Tipton et al.^30^	To identify and synthesize current knowledge and emerging concepts regarding systematic strategies that hospitals and health systems can implement to reduce length of stay (LOS), with emphasis on medically complex or vulnerable patients at high risk for prolonged LOS due to clinical, social, or economic barriers to timely discharge.	1. Published systematic reviews (SRs) of both randomized and nonrandomized primary studies were included if they met the inclusion criteria 2. Certain methodologic standards, such as providing search criteria, explicit inclusion/exclusion criteria, and risk‐of‐bias assessment.	1. SRs were excluded if they focused solely on patients undergoing nonemergent elective procedures or exclusively set in intensive care units, 2. Emergency departments, or managed or implemented by entities external to the hospital setting, such as community organizations. 3. Interventions not intended or expected to reduce LOS were not evaluated. 4. Systematic reviews were also excluded if they did not include LOS data. 5. Finally, we excluded SRs of primary studies that were either conducted solely outside the United States or if 50 percent or more of the studies reporting hospital LOS were conducted outside the United States.
Goncalves‐Bradley et al.^19^	To assess the effectiveness of planning the discharge of individual patients moving from the hospital	NA	NA
